# Abdominal Septic Shock – Endotoxin Adsorption Treatment (ASSET) – endotoxin removal in abdominal and urogenital septic shock with the Alteco® LPS Adsorber: study protocol for a double-blinded, randomized placebo-controlled trial

**DOI:** 10.1186/s13063-016-1723-4

**Published:** 2016-12-08

**Authors:** Miklos Lipcsey, Jyrki Tenhunen, Jan Sjölin, Robert Frithiof, Stepani Bendel, Hans Flaatten, Rafael Kawati, Anne Kuitunen, Tor Inge Tønnessen, Sten Rubertsson

**Affiliations:** 1 Hedenstierna Laboratory, Department of Surgical Sciences/Anaesthesiology and Intensive Care Medicine, Uppsala University, Uppsala, Sweden; 2 Department of Surgical Sciences/Anaesthesiology and Intensive Care Medicine, Uppsala University, Akademiska sjukhuset, 751 85 Uppsala, Sweden; 3 Section of Infectious Diseases, Department of Medical Sciences, Uppsala University, Uppsala, Sweden; 4 Department of Intensive Care, Kuopio University Hospital, Kuopio, Finland; 5 Department of Clinical Medicine, Haukeland University Hospital, UiB, Bergen, Norway; 6 Critical Care Medicine Research Group, Tampere University Hospital, PO Box 2000, 33521 Tampere, Finland; 7 Division of Emergencies and Critical Care, Oslo University Hospital and Institute of Clinical Medicine, 0450 Oslo, Norway

**Keywords:** Septic shock, Endotoxins, Hemoperfusion, Gram-negative bacteria

## Abstract

**Background:**

Severe sepsis and septic shock are common in intensive care and carry high mortality rates. In patients with Gram-negative infections, early and extensive removal of endotoxin may limit the inflammatory response that characterizes septic shock. The Alteco® LPS Adsorber (hereafter referred to cited as the lipopolysaccharide (LPS) Adsorber) can be used for endotoxin removal and attenuate the deleterious inflammatory and clinical responses seen in septic shock.

**Methods/design:**

The Abdominal Septic Shock – Endotoxin Adsorption Treatment (ASSET) trial is a pilot study investigating the feasibility and safety of LPS Adsorber therapy. This pilot, multicenter, stratified, parallel, double-blinded, randomized, phase IIa, feasibility clinical investigation will be performed in five Scandinavian intensive care units. Thirty-two subjects with early septic shock and organ failure, following adequate resuscitation, will be randomized to receive either: extracorporeal veno-venous hemoperfusion therapy with the LPS Adsorber or veno-venous hemoperfusion therapy with a placebo adsorber (without active LPS-binding peptide). Patients will be stratified by infection focus such that 20 subjects with an abdominal focus (stratum A) and 12 subjects with a urogenital focus (stratum B) will be included in a parallel design. Thereafter, an interim analysis will be performed and an additional 12 patients may be included in the study. The study is designed as adaptive a priori: the patients from this study can be included in a later phase IIb study. The aim of the study is to investigate the feasibility of LPS Adsorber therapy commenced early in the time-course of septic shock. The primary endpoint will be a characterization of all reported unanticipated serious adverse device effects and anticipated serious adverse device effects. Secondary outcomes are decrease in endotoxin plasma concentration, impact on clinical outcome measures and impact on inflammatory response by LPS Adsorber therapy, as well as detailed description of the relevant mediators bound to the LPS Adsorber. Recruitment of patients will start in September 2015.

**Discussion:**

The ASSET trial will give insight into the feasibility and safety of this LPS Adsorber therapy and preliminary data on its potential clinical effects in septic shock. Moreover, this pilot trial will provide with necessary data for designing future studies.

**Trial registration:**

ClinicalTrials.gov Identifier NCT02335723. Registered on 28 November 2014.

## Background

Although severe sepsis and septic shock are common in intensive care and carry high mortality rates no specific treatment has been established to counteract the associated inflammatory response. Current management may be considered supportive only and consists mainly of effective antimicrobial therapy, removal or drainage of infections (source control) and support of failing organs [1]. Therefore, development of new treatment methods for sepsis and septic shock is crucial for medical, humanitarian and health-economic reasons.

Endotoxins are molecules found in the outer membrane of the cell wall in Gram-negative bacteria. They are potent activators of the inflammatory system through the innate immune system and are presumably the key triggers of the systemic inflammatory response [2–4]. Hence, attenuating the effect of endotoxins seems to be a logical and desirable strategy in the treatment of severe sepsis and septic shock. However, so far the use of anti-endotoxin strategies has been disappointing [5–8]. On the other hand, adsorber therapy was proven beneficial in early sepsis patients due to intra-abdominal infections and added substantially to improved outcome in conjunction with conventional interventions. Improved outcome was associated with reduced endotoxin levels in blood [9, 10]. Therefore, it is reasonable to hypothesize that using adsorption membranes in the treatment of selected Gram-negative septic shock in the early phase, with a strictly defined time-frame since the onset of clinical symptoms, may offer therapeutic benefits. Several extracorporeal endotoxin-removal devices, based on various endotoxin-binding mechanisms, have been investigated with diverging results [9, 11, 12]. Currently, at least one trial is investigating the effect of endotoxin removal with polymyxin B cartridges [13].

Another endotoxin removal device based on a different concept is a novel, high-capacity adsorber system (from Alteco® Medical AB, Lund, Sweden) with a high-endotoxin-affinity-peptide endotoxin-binding mechanism (hereafter referred to as the lipopolysaccharide (LPS) Adsorber) that has been clinically available for some years. This adsorber putatively binds endotoxin and thus decreases endotoxin load in patients with Gram-negative sepsis. Case reports and series from patients with septic shock suggest that endotoxin removal could be achieved in clinical practice with LPS Adsorber [14, 15]. However, no randomized controlled trial has investigated the potential benefits of this LPS Adsorber. LPS Adsorber is described as biocompatible and no specific side effects related to it have been reported.

We hypothesized that in patients with Gram-negative infections, early and extensive removal of endotoxin may limit the inflammatory response that characterizes septic shock. This LPS Adsorber can be used for endotoxin removal and, in this way, add to the currently available strategies for sepsis treatment.

The present clinical investigation aims to collect information on the feasibility and safety of LPS Adsorber therapy in patients with septic shock of presumed abdominal or urogenital origin. Secondary aims are to report the performance of the LPS Adsorber. Specifically, we wish to study the extent of endotoxin removal by the LPS Adsorber, its effects on organ failure development and the extent of organ support, as well as its possible impact on the inflammatory response.

This trial should contribute to new knowledge since it investigates a high-capacity peptide-binding-based endotoxin removal that can be run for longer periods and at higher blood flow that other endotoxin adsorbers. Moreover, unlike in other device trials, the LPS Adsorber will be compared to an identical LPS Adsorber cartridge without active LPS-binding peptide. Thus, a double-blinded randomized controlled trial will be possible to perform.

Finally, the ultimate objective of this pilot investigation is to provide the necessary information on the magnitude of the clinical effects of the LPS Adsorber, thus providing the basis for further investigations in a larger population.

## Methods/design

This is a pilot, multicenter, stratified, parallel, double-blinded, randomized, phase IIa, feasibility clinical investigation and is reported according to the Standard Protocol Items: Recommendations for Interventional Trials (SPIRIT) guidelines [16]. Subjects will be enrolled in accordance with an adaptive design, with an interim analysis allowing enrollment of additional patients until an adequate sample size is attained, to show the safety and the feasibility or lack thereof of LPS Adsorber therapy in the target patient population.

The study will be conducted in five general intensive care units (ICU) in Uppsala, Sweden, Bergen and Oslo, Norway, and Tampere and Kuopio, Finland. These ICUs will be screened around the clock to identify potential patients. Initially, 32 subjects admitted to the ICU with confirmed septic shock will be stratified in accordance with the origin of their suspected endotoxemia, i.e., 20 septic shock subjects with an abdominal focus (stratum A) and 12 subjects with a urogenital focus (stratum B) will be randomly allocated to the active LPS Adsorber group (Interventional Medical Device group, IMD group): current best practice in combination with LPS Adsorber therapy *or* placebo device group (control group): current best practice in combination with placebo adsorber therapy (identical LPS Adsorber cartridge without active LPS-binding peptide). The random allocation to either therapy arm in each stratum will be performed in a 1:1 ratio.

**Fig. 1:**
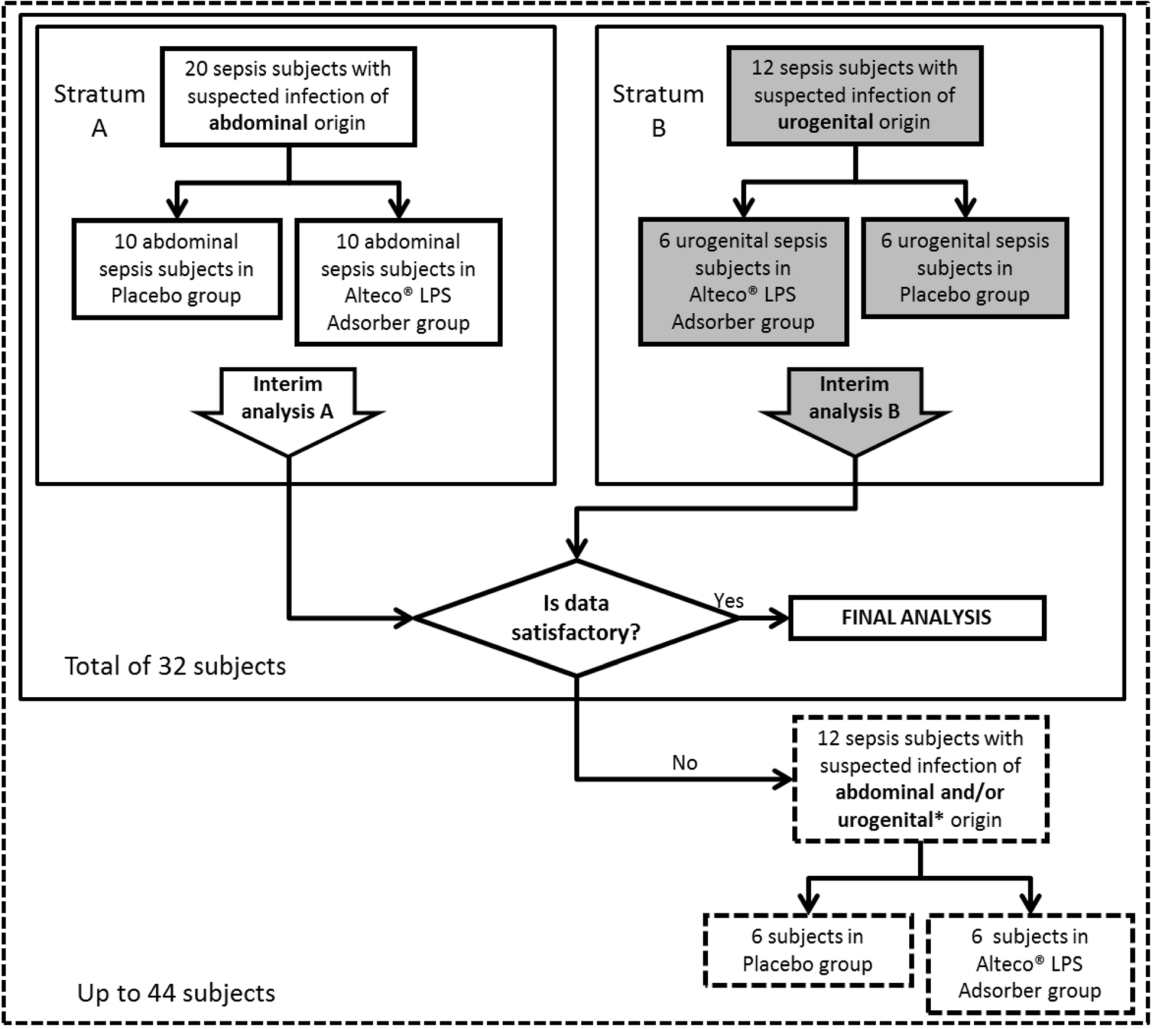
Enrollment plan for each stratum and interim analysis. *Optional enrollment of sepsis subjects (of abdominal, urogenital or both origins) after interim analysis

An interim analysis will be performed after all subjects in their corresponding stratum have completed their participation in the investigation to reveal whether 12 additional subjects are required. The enrollment plan in the clinical investigation is summarized in Fig. 1.

The study has an adaptive design and thus the investigators have made an a-priori decision to include the patients from this study in a later phase IIb study aiming to investigate and report possible outcome benefits of the therapeutic intervention.

### Inclusion criteria

Upon enrollment (i.e., pretreatment phase), subjects admitted to the ICU with suspected endotoxemia will be screened for fulfillment of the “Illness severity criteria” confirming early stage severe sepsis.

Within 6 h of enrollment, subjects who also fulfill the “Treatment criteria” confirming septic shock will be eligible for randomization.

The subjects have to meet all of the following criteria to be eligible to enter the clinical investigation:

#### Illness severity criteria

At enrollment, subjects must meet the inclusion criteria #1 through #5 listed below to be eligible to enter the clinical investigation:

1. Subjects must have suspected infection of abdominal or urogenital origin for which the subject is receiving intravenous antimicrobial therapy
2. Subjects must have systemic inflammatory response syndrome (SIRS), i.e., they must meet at least two of the criteria defined below during the 36 h prior to clinical investigation entry:
  - Abnormal body temperature:
    i.) Fever: core temperature (measured via rectum, urinary bladder, central catheter) above 38 ° C (100.4 ° F), *or*
    ii.) Hypothermia: core temperature (measured via rectum, urinary bladder, central catheter) below 36 ° C (96.8 ° F)
  - Heart rate above 90 beats/min
  - Spontaneous respiratory rate above 20 breaths/min *or* PaCO_2_ < 32 mmHg (4.3 kPa) *or* concurrent use of mechanical ventilation for an acute process
  - White blood cell count (WBC) of >12,000 cells/mm^3^ or <4000 cells/mm^3^ *or* a >10% increase in immature neutrophils
3. Subjects must have clinical signs consistent with organ dysfunction, i.e., they must meet at least one of the following criteria during the 6 h prior to clinical investigation entry:
4. Subjects, independent of gender, must be aged 18 years or older
5. Subjects or legally acceptable representatives, as appropriate, are willing and able to provide signed informed consent

#### Treatment criteria

6. Prior to randomization, subjects must meet all inclusion criteria (#7 through #11) listed below to be assigned to a treatment group:
7. Subjects must have septic shock according to the modified septic shock criteria, i.e., Illness severity criteria #1 through #3 are still fulfilled
8. Subjects must score a Sequential Organ Failure Assessment (SOFA) score of 10 or higher, *and* a Simplified Acute Physiology Score (SAPS II) of 58 or higher
9. Appropriate vascular access must have been obtained
10. Subjects must have received ≥30 mL/kg of intravenous fluid within the 6 h prior to randomization
11. Subjects must have a continuous requirement for vasopressor support (at least one of the vasopressors must be given as listed below) for at least 2 h prior to randomization to maintain mean arterial pressure (MAP) >65 mmHg or systolic arterial pressure >90 mmHg with reasonable attempts made to wean the subject from vasopressor support. Careful evaluation is made so that vasopressor dependency is *not only* related to sedation. Required vasopressor support includes at least one of the following vasopressors administered as described below (and allows for combination therapy where other vasopressors are given with modified administration regimen):
  - Norepinephrine ≥0.07 μg/kg/min
  - Dopamine ≥10 μg/kg/min
  - Phenylephrine ≥35 μg/kg/min
  - Epinephrine ≥0.07 μg/kg/min
  - Vasopressin ≥0.03 units/min

12. Subjects must be able to initiate the clinical investigation intervention within 12 h of fulfillment of the Illness severity criteria

### Exclusion criteria

Subjects who meet any of the exclusion criteria listed below will *not* be permitted to enter the clinical investigation:

- Sepsis-induced organ dysfunction for longer than 12 h prior to the time point for achieving “Illness severity criteria fulfilled”
- Vasopressor therapy (at any dose) for longer than 8 h (not including the time spent in the operation theatre) prior to the start of the therapy with the investigational device
- Preexisting irreversible medical conditions such as:
  - ○ Poorly controlled neoplasms or hematologic disease (i.e., indication of disseminated cancer outside the suspected primary tumor and hematologic disease not in remission)
  - ○ End-stage cardiac disease
  - ○ Cardiac arrest requiring cardiopulmonary resuscitation or with pulseless electrical activity or asystole within the past 7 days
  - ○ End-stage lung disease; end-stage liver disease
  - ○ HIV/AIDS with known end-stage processes
  - ○ Other uncorrectable medical condition(s) deemed by the clinical investigator to hinder the subject to adhere to the fulfillment of the activities described in the Clinical Investigation Plan (CIP)
- Extreme illness, i.e., subject is moribund and death is perceived to be imminent (within 24 h)
- Recent or current participation (≤30 days) in another interventional sepsis trial
- Recent or current treatment (≤30 days) with an adsorption product, including the LPS Adsorber
- Treatment with an investigational medicinal product for any indication within the last 30 days before enrollment in the clinical investigation
- Pregnancy
- Contraindications to the use heparin or protamine, as in case of, but not limited to:
  - ○ Prior heparin-induced thrombocytopenia II (HIT II)
  - ○ Prior intracranial bleeding of any cause up to 90 days before inclusion in this clinical investigation
  - ○ Prior neurosurgery up to 90 days before inclusion in this clinical investigation. Prior hemorrhagic stroke up to 90 days before inclusion in this clinical investigation
  - ○ Fish allergy
- Other abdominal inflammatory conditions such as, but not limited to:
  - ○ Suspected pancreatitis (serum amylase (s-amylase) level at least three times the upper limit of normal (ULN))
  - ○ Suspected fulminant hepatitis
  - ○ Suspected gastric/duodenal ulcer-related gastrointestinal perforation
- Perforation of hollow organ linked to trauma within 48 h before enrollment in the clinical investigation
- Laparotomy reveals isolated gastric ulcer
- Subjects and/or their immediate family are directly affiliated to investigative site personnel in this clinical investigation (immediate family is defined as a spouse, parent, child or sibling, whether biological or legally adopted)

### Intervention

Upon enrollment (i.e., pre-treatment phase), subjects admitted to the ICU with suspected endotoxemia will be screened for fulfillment of the “Illness severity criteria” confirming the early stages of severe sepsis. Within 6 h of enrollment, subjects who also fulfill the “Treatment criteria” confirming septic shock will be eligible for randomization. Randomization to either of the treatment groups will be performed as close as possible to the start of treatment with the LPS Adsorber or placebo device.

Treatment with the LPS Adsorber or placebo device must be initiated within 6 h (day 1) following fulfillment of the “Treatment criteria” and given for 6 h. A second device treatment will be performed 24 h after the end of the first device treatment on day 2, as long as there is no evidence that treatment with the investigational device will not be beneficial or will indicate an unnecessary risk for subjects (for example, if the subject is free of vasopressor support).

The treatment with the LPS Adsorber or placebo can be discontinued at any time on the decision of the attending physician. At least one 2-h treatment session is required to fulfill the treatment protocol.

**Fig. 2:**
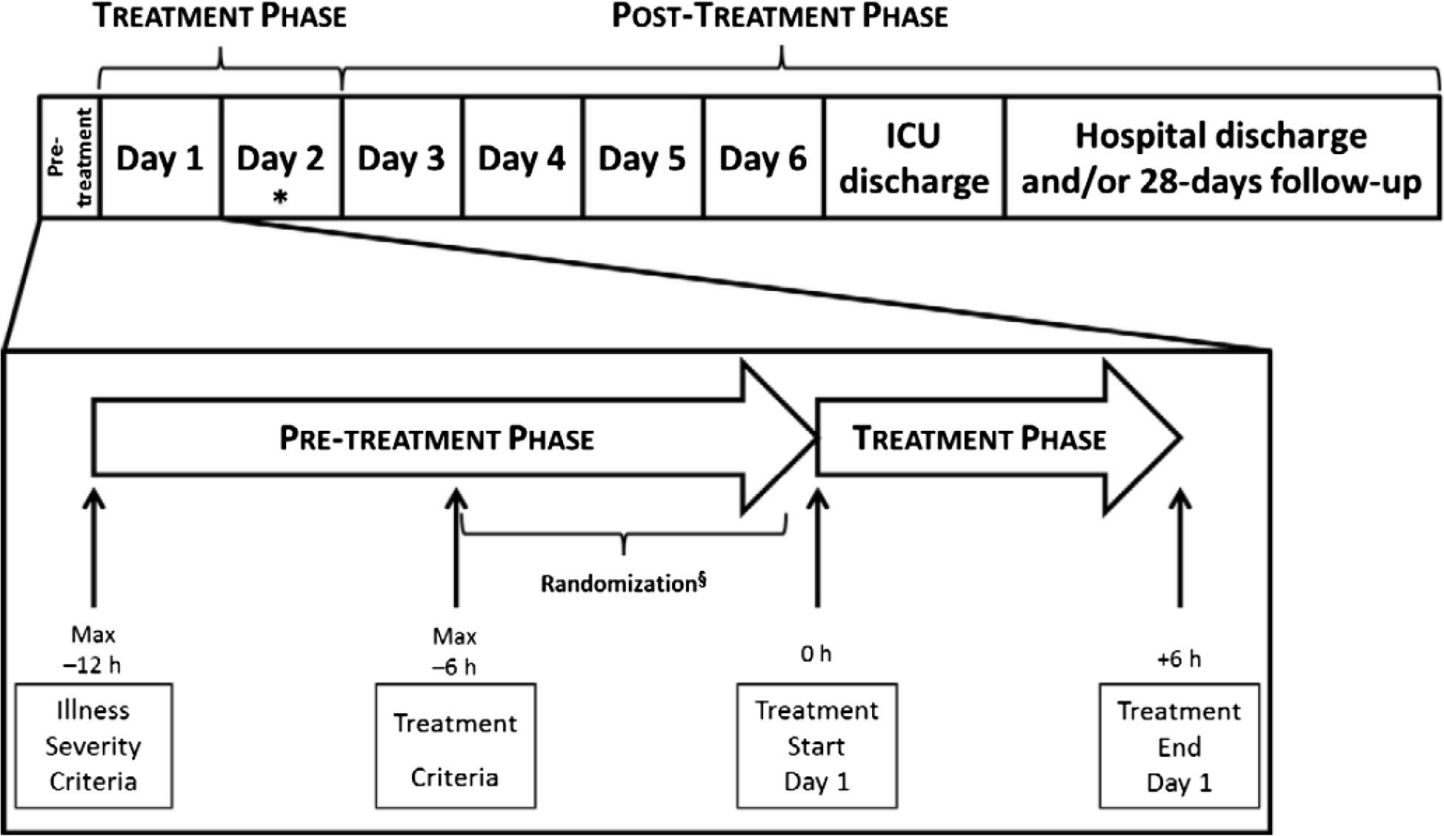
Overall clinical investigational design. *Treatment with IMD or placebo device will be repeated on Day 2, i.e. 24 h after end of treatment on day 1. ^§^ Randomization will be performed as close as possible to start of treatment start (time-point O h) on Day 1

After treatment with the LPS Adsorber or placebo is finalized (post-treatment phase), subjects will be monitored until they are ready for ICU discharge or deceased. Discharge from hospital and mortality at 28 days since enrollment will also be collected. Figure 2 and Table 1 summarize the participant timeline of the clinical investigation.

**Table 1:** The timeline of the study according to the Standard Protocol Items: Recommendations for Interventional Trials (SPIRIT) guidelines

Assessments	Pre-treatment phase		Treatment phase						Post-treatment phase		
	Screening		Day 1			Day 2			Days 3–6	ICU discharge	28-day follow-up
	Illness criteria	Treatment criteria	Baseline	Following start of treatment 1	End of treatment 1	Prior to treatment 2 start	Start of treatment 2	End of treatment 2			
	up to −12 h	up to −6 h	Before 0 h	0 h	6 h	Before 30 h	0 h	6 h	~48–120 h		Max 28 days
Enrollment											
Informed consent	X										
Demography, medical history	X									X	
Vital signs, organ support Assessment	X		X	X		X	X		X		
Physical examination	X		X	X	X	X	X	X	X		
Laboratory assessments	X		X	X	X	X	X	X	X		
Illness severity scoring	X	X	X	X	X	X	X	X	X	X	X
Eligibility criteria #1	X										
Eligibility criteria #2		X									
Randomization		X									
Intervention											
Investigational/placebo device treatment				X			X				
Assessments											
Laboratory assessments	X		X	X	X	X	X	X	X		
Illness severity scoring	X	X	X	X	X	X	X	X	X	X	X
Prior or concomitant therapy	X	X	X	X	X	X	X	X	X	X	
AEs			X	X	X	X	X	X	X	X	
ADEs			X	X	X	X	X	X	X	X	
Device deficiencies			X	X		X	X				
ICU-LOS										X	
Hospital-LOS											X
Mortality (ICU and 28-day)										X	X

*ADEs* adverse device effects, *AEs* adverse events, *ICU* intensive care unit, *LOS* length of stay

### Concomitant therapy

Medication, which is considered necessary for the subjects’ safety and wellbeing, may be given at the discretion of the investigator. Relevant medications or other interventions include, but are not limited to current best practice for the treatment of septic shock as described in the Surviving Sepsis Campaign guidelines [1].

Administration of concomitant medication/therapy during the conduct of the investigation may lead to withdrawal of the subject from the clinical investigation. Prohibited medications/therapies include:

- Medications without a marketing approval in the country of the site
- Colloids (with the exception of albumin) for use in fluid resuscitation
- The use of renal replacement therapy (RRT) concomitantly with the use of the LPS Adsorber or placebo device is allowed between device therapies on day 1 and day 2, and after the end of therapy phase on day 2

### Blinding

This is a double-blinded study. Data is collected by an independent contract research organization (TFS Trial Form Support AB, Lund, Sweden). Neither health care providers nor Steering Committee members, investigators or statisticians will have access to group allocation of subjects. The LPS Adsorber contains a designed synthetic peptide developed for endotoxin adsorption. The placebo comparator device differs from the LPS Adsorber only in that no peptide component (i.e, active component) has been attached to the matrix. Both the LPS Adsorber and the placebo device will be relabelled for the purpose of this clinical investigation. The investigational site(s) will be provided with emergency code break envelopes for all subjects.

### Randomization

Screening will be web-based. Patients are randomized to either the LPS Adsorber or placebo adsorber arms by being treated with a blinded cartridge on site starting with the lowest serial number. The abdominal focus (stratum A) and the urogenital focus (stratum B) patients are kept apart by different serial numbers. This results in double-blinding, stratification by sepsis focus and randomization in blocks.

### Primary objective

The primary objective of this clinical investigation is to investigate the feasibility, safety and possible benefits of the LPS Adsorber in treating patients with septic shock with presumed endotoxemia of abdominal or urogenital origin.

### Secondary objective

The secondary objectives are:

- To investigate the ability of the LPS Adsorber to reduce levels of endotoxin in plasma during and directly after treatment with the investigational device
- To investigate the impact of the LPS Adsorber on clinical outcome
- To investigate the impact of the LPS Adsorber on the inflammatory response in septic shock
- To investigate the content bound to the LPS Adsorber in a subset of subjects after treatment end

### Endpoints

#### Primary endpoint

Primary endpoint is characterization of all reported unanticipated serious adverse device effects and anticipated serious adverse device effects.

#### Secondary endpoints

- Relative change from baseline in plasma endotoxin (p-endotoxin) levels during (i.e., at 2 h) and immediately after end (i.e., at 6 h) of therapy with device, on both day 1 and day 2
- Monitoring of clinical outcome parameters during stay at ICU:
  - ○ Relative change from baseline in SOFA score (total and organ specific) measured once daily and up to 120 h (or time for ICU discharge, whichever comes first) after start of therapy with device
  - ○ Relative change from baseline in the monitoring of renal function
  - ○ Relative change from baseline in the monitoring of liver function
  - ○ Relative change from baseline in circulatory support
  - ○ Relative change from baseline in respiratory support
  - ○ ICU mortality
  - ○ ICU length-of-stay (LOS) up to 28 days
- Monitoring of clinical outcome parameters during stay at hospital following ICU discharge:
  - ○ The total extension of renal support
  - ○ 28-day mortality
  - ○ Hospital-LOS up to 28 days
- Monitoring of inflammatory response biomarkers:
  - ○ Inflammatory triggers and markers
  - ○ Acute phase protein markers
  - ○ Plasma cytokines
- Determination of the molecular components extracted from blood circulation and captured in the LPS Adsorber
- Characterization of all reported adverse events (regardless of attribution), adverse device effects and device deficiencies

### Participant withdrawal

Participation in the clinical investigation is voluntary and subjects (or their legally accepted representatives) may discontinue their participation at any time.

Subjects may be withdrawn from investigational treatment and assessments at any time if deemed necessary by the investigator.

Potential reasons for withdrawal of subjects from this clinical investigation are:

- Screening failure (if any inclusion criterion is not fulfilled or if any exclusion criterion is fulfilled)
- The decision of a subject or legally acceptable representative(s), as appropriate, to withdraw from the clinical investigation (including if the subject withdraws informed consent)
- Administration of concomitant medication or procedure prohibited by this investigation plan
- Need of other intervention, for medical reasons, that leads to the interruption of the sepsis treatment. Examples:
  - ○ Imminent need of surgery
  - ○ Imminent need of continuous renal replacement therapy (CRRT), defined as
    - Plasma potassium >6.5 mmol/L despite other therapeutic options
    - Life-threatening fluid overload
  - ○ Computed tomography (CT) scan
- Pregnancy
- Subject is lost to follow-up

### Severe adverse reactions

The LPS Adsorber is a class IIa medical device and does not include any drugs or toxic substances that enter the blood circulation. All components in the LPS Adsorber are biocompatible. The capturing-peptide component is nontoxic, and cannot be released during rinsing or therapy procedures as it is covalently bound to the matrix. The LPS Adsorber is part of an extracorporeal hemoperfusion system which is supplied sterile and only for single use.

All serious adverse events and serious adverse device effects are promptly reported by the investigation site to Clinical Drug Safety Support at the contract research organization (TFS Trial Form Support AB, Lund, Sweden) within 24 h of learning about the event, regardless of the time that may have elapsed from the time the event occurred and these will be evaluated and recorded.

### Amendments

Protocol amendments will be made by the Steering Committee and submitted to the ethical boards in each participating country for approval. The protocol presented is version 5 from 2 March 2016.

### Acquisition of data

An eCRF (electronic Case Report Form) system will be used. Data validation and data queries will be handled by the contract research organization‘s Data Management Team (TFS Trial Form Support AB, Lund, Sweden).

### Data handling and record keeping

A monitor from the contract research organization (TFS Trial Form Support AB, Lund, Sweden) will perform monitoring activities at the assigned clinical investigational site(s) in accordance with the monitoring plan. Management and storage of data will be handled by the contract research organization.

### Data ownership and publication policy

The investigators and the sponsor will be joint owners of all materials and all results. Any publication shall be subject to the prior review and approval of the sponsor, such approval is not to be unreasonably withheld. The sponsor shall not have a right for veto in any correct factual part of any planned publication.

### Statistics

#### Power calculation

Since this is a pilot study, no sample size calculation was possible or has been performed. The sample size was chosen for practical reasons, and aimed to allow recruitment of all subjects in the first enrollment period (i.e., stratum A and stratum B) within a year, and within six additional months for subjects in the second enrollment period. Data from this pilot investigation will provide information for designing future studies with the LPS Adsorber.

#### Endpoints

No primary performance variables will be collected in the present clinical investigation. The statistical analyses of secondary endpoints will be descriptive.

#### Analysis sets

The following analysis sets will be used for the statistical analysis:

The Full Analysis Set (FAS) will consist of all randomized subjects who were treated with the investigational device or placebo device for at least 2 h on day 1.

The Per Protocol Set (PPS) will consist of subjects from the FAS who completed at least 2 h of therapy with the investigational device or the placebo device on day 1 and on day 2. Additionally, the subjects in the PPS will not have any major CIP violations that will affect the evaluation. Major CIP violations will include but are not limited to the following:

- Nonfulfillment of all inclusion criteria
- Fulfilling at least one exclusion criteria

The FAS is considered as the primary analysis dataset, and will be used for all performance analyses. The analysis(es) that will be repeated using the PPS will be further described in the SAP. Major discrepancies between the results from the FAS and the PPS analyses will be compared and discussed.

The Safety Set will consist of all randomized subjects who started therapy. Safety summaries will be performed on the Safety Set.

#### Interim analysis

An interim analysis will be performed after all subjects in their corresponding stratum have completed their participation in the investigation to assess whether 12 additional subjects are required.

## Discussion

To date, there is no specific therapy for the inflammatory response induced by bacteria in sepsis. Given its role in triggering inflammatory responses, endotoxin could play a central role in inducing organ failure in Gram-negative sepsis. Endotoxin removal with an endotoxin-specific, high-affinity, high-capacity adsorber system is, therefore, an attractive therapeutic option. The LPS Adsorber used in this study fulfills these characteristics.

The key goal of this study is to identify patients who might benefit from endotoxin removal. This goal is achieved by including severely ill septic shock patients with presumed endotoxemia and initiating endotoxin removal therapy early. The objective of the ASSET study is to investigate the safety and feasibility of LPS Adsorber therapy and to provide data for further studies.

### Trial status

Recruitment of patients started in September 2015.

## Abbreviations

ALT: Alanine aminotransferase
AST: Aspartate aminotransferase
CIP: Clinical investigation plan
CRRT: Continuous renal replacement therapy
CT: Computed tomography
eCRF: Electronic Case Report Form
HIT II: Heparin-induced thrombocytopenia II
ICU: Intensive care unit
IMD: Interventional Medical Device
LOS: Length-of-stay
MAP: Mean arterial pressure
RRT: Renal replacement therapy
SAPS II: Simplified Acute Physiology Score
SIRS: Systemic inflammatory response syndrome
SOFA: Sequential Organ Failure Assessment
